# Optical Coherence Tomography Angiography Changes in Macular Area in Patients with Proliferative Diabetic Retinopathy Treated with Panretinal Photocoagulation

**DOI:** 10.3390/biomedicines11123146

**Published:** 2023-11-26

**Authors:** Irini Chatziralli, Eleni Dimitriou, Chrysa Agapitou, Dimitrios Kazantzis, Petros Kapsis, Nick Morogiannis, Stylianos Kandarakis, George Theodossiadis, Panagiotis Theodossiadis

**Affiliations:** 1 2nd Department of Ophthalmology, Attikon Hospital, National and Kapodistrian University of Athens, 12462 Athens, Greece; 2 1st Department of Ophthalmology, National and Kapodistrian University of Athens, 12462 Athens, Greece

**Keywords:** proliferative diabetic retinopathy, microvasculature, vessel density, OCTA, foveal avascular zone, panretinal photocoagulation

## Abstract

Background: The purpose of this study was to investigate the changes in macular microvasculature using optical coherence tomography angiography (OCTA) in association with functional changes in patients with proliferative diabetic retinopathy (PDR) treated with panretinal photocoagulation (PRP) with a follow up of 12 months. Methods: The participants in this study were 28 patients with PDR and no macular oedema, who were eligible for PRP. All participants underwent best-corrected visual acuity (BCVA) measurement, optical coherence tomography (OCT), and OCT angiography (OCTA) at baseline (before treatment) and at months 1, 6, and 12 after the completion of PRP treatment. The comparison of OCTA parameters and BCVA between baseline and months 1, 6, and 12 after PRP was performed. Results: There was a statistically significant decrease in foveal avascular zone (FAZ) area at months 6 and 12 of the follow-up period compared to baseline (*p* = 0.014 and *p* = 0.011 for month 6 and 12, respectively). Of note is that FAZ became significantly more circular 6 months after PRP (*p* = 0.009), and remained so at month 12 (*p* = 0.015). There was a significant increase in the mean foveal and parafoveal vessel density (VD) at all quadrants at the superficial capillary plexus (SCP) at month 6 and month 12 after PRP compared to baseline. No difference was noticed in VD at the deep capillary plexus (DCP) at any time-point of the follow up. BCVA remained the same throughout the follow-up period. Conclusions: At months 6 and 12 after PRP, foveal and parafoveal VD at SCP significantly increased compared to baseline, while the FAZ area significantly decreased and FAZ became more circular.

## 1. Introduction

Diabetes mellitus (DM) is a global epidemic which is estimated to affect about 785 million people in 2045 [1]. Diabetic retinopathy (DR) is a microvascular complication of DM and a leading cause of blindness worldwide, especially in the working-age population [2]. Diabetic retinopathy is classified as either non-proliferative (NPDR) and proliferative (PDR). NPDR is characterized by structural changes in retinal capillaries, including microaneurysms, haemorrhages, exudates, cotton wool spots, venous beading or looping, intraretinal microvascular abnormalities (IRMA), and retinal non-perfusion. The latter may trigger the development of neovascularization either on the disc (NVD) or elsewhere on the retina (NVE), leading to PDR [2].

Panretinal photocoagulation (PRP) using an argon laser is considered the “gold standard” for the treatment of PDR, since it has been proven to reduce the risk of severe visual loss or need for vitrectomy by over 50% in high-risk PDR patients at four-year follow up [3]. Regarding the mechanism by which PRP helps in the regression of neovascularization, there are several theories. Firstly, it was suggested that the destruction of the retina using laser thermal ablation can lead to the reduction in metabolic demand, or, in simple terms, the diseased retina could be debulked while also achieving the suppression of vascular endothelial growth factor (VEGF) production [4,5]. Additionally, the thinning of the retina due to photocoagulation would facilitate increased oxygen diffusion from the choroid to the vitreous and consequent improvement in inner retinal oxygenation via diffusion through the vitreous. Moreover, the stimulation of biological factors, such as heat shock proteins, might result in the improvement of the disease *per se* [5]. However, PRP may disrupt the blood–retinal barrier, leading to alterations in the retinal microvasculature and haemodynamics [6]. Specifically, a reduction in ocular blood flow velocities in the ophthalmic artery, central retinal artery, and central retinal vein has been reported after PRP in patients with DR using Doppler measurement or laser speckle flowgraphy [7,8,9]. 

On the other hand, advances in retinal imaging and the introduction of optical coherence tomography angiography (OCTA) allow the non-invasive visualization of the retina, also providing quantitative data, including measurements of the vessel density (VD) and the foveal avascular zone (FAZ) [10]. There have been a few studies reporting changes in circulatory distribution and vascular parameters in the macular area after PRP in patients with severe NPDR and PDR, with controversial results [11,12,13,14]. Russell et al. showed that retinal perfusion did not change significantly 3 months after PRP in 15 patients with PDR [11] in a prospective study using ultra-widefield fluorescein angiography (FA), the findings of which were in line with other investigators, who found similar results at months 3–6 after PRP using OCTA [12,13]. On the contrary, Fawzi et al. reported an increase in the flow metrics of all capillary layers in the macula using OCTA 3–6 months after PRP in 10 patients with PDR, suggesting an overall re-distribution of blood flow to the posterior pole following PRP in patients with PDR [14]. 

However, the as yet published studies have short follow ups of 1–6 months and small study sample, ranging from 6 to 15 patients, with an average age of patients between 46 and 63 years [11,12,13,14]. In light of the above, the purpose of this prospective study was to evaluate the changes in macular microvasculature using OCTA in association with visual changes in patients with PDR treated with PRP in a 12-month follow-up study. 

## 2. Materials and Methods

Participants in this prospective study were 28 patients with type 2 DM and PDR. All patients were recruited, diagnosed, treated, and followed up at 2nd Department of Ophthalmology, National and Kapodistrian University of Athens, Athens, Greece between September 2020 and September 2022. The study was in adherence with the tenets of Helsinki Declaration and was approved by the Institutional Review Board of Attikon General Hospital (Ref: 291/2020). Written informed consent was obtained from all patients after explaining them the protocol of the study. 

All participants were treatment-naïve patients with high-risk PDR, and did not have diabetic macular oedema. PDR was diagnosed through detection of retinal neovascularization on dilated fundoscopy, and confirmed with FA when there was uncertainty regarding its presence. In case of bilateral PDR, right eye was included to avoid selection bias. Eyes with other retinal diseases, glaucoma, intraocular pressure (IOP) ≥ 18 mmHg, ocular inflammation, spherical equivalent ≥ 6 diopters, axial length ≥ 26.5 mm, significant media opacity to preclude adequate imaging, vitreous haemorrhage, previous pars plana vitrectomy, previous cataract surgery within the last 6 months, trauma, and previous treatment for DME or DR were excluded. All patients were followed up for at least 12 months. 

At baseline, we recorded patients’ information, including age, gender, diabetes duration, presence of hypertension, dyslipidaemia, and glycated haemoglobin (HbA1c). In addition, at baseline, all participants underwent a thorough ophthalmic examination, including best-corrected visual acuity (BCVA) measurement by means of ETDRS charts, slit-lamp examination, IOP measurement, dilated fundoscopy, spectral domain optical coherence tomography (SD-OCT), OCTA, and fundus photography using HOCT-1F All-in-one (Huvitz, Korea). In cases where FA was needed, it was performed using Heidelberg Spectralis (Spectralis HRA+OCT, Heidelberg, Germany). BCVA assessment, slit-lamp examination, dilated fundoscopy, SD-OCT, and OCTA were repeated at each follow-up visit, while FA was performed at physician’s discretion. BCVA was converted to LogMAR for statistical purposes.

All patients were treated with PRP, and were examined at month 1, 6, and 12 after treatment. Panretinal photocoagulation was carried out using Visulas Green Laser 532 nm (Carl Zeiss Meditec AG, Jena, Germany) in 3 sessions with an interval of 1 week in between, with a total number of about 2000–2500 burns. The covered area extended from optic disc by a diameter of about one disc, and to posteriorly just outside the arcades, reaching as far as possible into the periphery, while in the macula, the temporal demarcation border was defined as about 1.5 times the disc-to-fovea distance positioned temporal to the fovea. Laser settings were 200 μm spot size with pulse duration of 200 ms, and power of 200–250 mW, while the laser burns were spaced one laser spot size apart. No complications were reported during or after PRP in our study sample. 

### 2.1. Optical Coherence Tomography (OCT) and OCT Angiography Protocol

**Figure 1:**
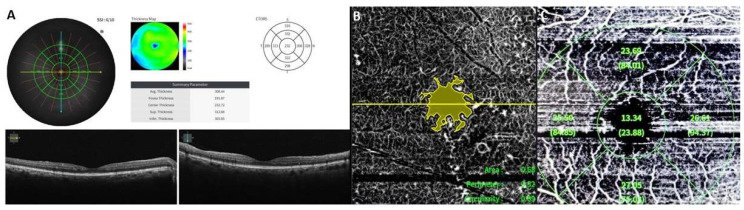
(**A**) Optical coherence tomography (OCT) of a patient with diabetes mellitus and no diabetic macular oedema, depicting the protocol of OCT imaging; (**B**) OCT–angiography of the same patient showing the foveal avascular zone (FAZ) area measurement, FAZ perimeter, and FAZ circularity, as well as vessel density measurement (**C**), according to the OCT–angiography imaging protocol.

The HOCT-1F All-in-one uses a diode beam source of 840 nm with a scanning speed of 68,000 axial scans per second, and has an intelligent fixation system that avoids artifacts from ocular movements. For SD-OCT, we used the macular radial 3D OCT scan pattern with 9 mm scan range, 512 A-scan points, and 96 B-scan lines, enabling “fine” OCT sensitivity. An area of 6 × 6 mm centred in the fovea was analysed in agreement with the Early Treatment Diabetic Retinopathy Study (ETDRS) map [15]. The macula was divided into nine regions and three concentric rings (the 1 mm diameter at foveal centre, the inner ring at 3 mm diameter, and the outer ring at 6 mm diameter), which were subdivided into four subfields (superior, temporal, inferior, and nasal) (Figure 1A). All measurements were obtained using the automated segmentation algorithms from the OCT unit. The central subfield thickness (CST) was defined as the mean thickness of the neurosensory retina in the central 1 mm diameter determined by the ETDRS map. In addition, the condition of ellipsoid zone and external limiting membrane was assessed at an area of 1500 μm radius around the foveola.

Regarding OCTA, we used the “Macular Angio” protocol with 4.5 mm scan range, 384 A-scan points, and 384 B-scan lines. The OCTA analysis software calculated the foveal avascular zone (FAZ) area in mm^2^, the FAZ perimeter in mm, and the FAZ circularity ratio (Figure 1B) automatically, as well as the vessel density (VD) in foveal and parafoveal region (Figure 1C) in both superficial capillary plexus (SCP) and deep capillary plexus (DCP). SCP was defined as from internal limiting membrane (ILM) to inner plexiform layer (IPL), while DCP was defined as from IPL to outer plexiform layer (OPL). The foveal region was defined as a 1 mm ring centred on the fovea, and the parafoveal region was defined as the zone between the 1 mm and 3 mm concentric rings centred on the fovea. FAZ represents a region absent of capillaries at the centre of the fovea.

### 2.2. Statistical Analysis

Patients’ characteristics were presented using descriptive statistics. Mean ± standard deviation (SD) was used for continuous variables and counts for categorical variables. Normal distribution of data was analysed by the Kolmogorov–Smirnov test. Longitudinal comparisons for various parameters between baseline and months 1, 6, and 12 was performed, using ANOVA for repeated measures or McNemar test, as appropriate. 

Statistical analysis was conducted using IBM SPSS (Version 22.0, Chicago, IL, USA). A *p* value ≤ 0.05 was considered as statistically significant. However, for longitudinal multiple comparisons, the Bonferroni correction was adopted, as appropriate. Specifically, since 3 comparisons were performed (baseline versus month 1, month 6, and month 12), statistical significance was set to 0.05/3 = 0.017.

## 3. Results

A total of 28 patients (28 eyes) with treatment-naïve PDR were enrolled in the study. Table 1 shows the demographic and clinical characteristics of the study sample. The mean age of patients was 57.3 ± 8.9 years. A total of 15 patients (53.6%) were male and 13 (46.4%) were female. The mean duration of DM was 12.4 ± 5.4 years. The mean HbA1c at baseline was 7.8 ± 1.3%. Regarding comorbidities, 18 out of 28 patients (64.3%) had hypertension, and 12 patients (42.9%) Had dyslipidaemia, while 11 out of 28 patients (39.2%) were current smokers. Furthermore, 6 out of 28 patients (21.4%) were pseudophakic. 

At baseline, the mean BCVA was 0.41 ± 0.11 logMAR. There was no statistically significant difference in BCVA at month 1 (0.41 ± 0.13 logMAR, *p* = 0.030), month 6 (0.40 ± 0.12 logMAR, *p* = 0.103), and month 12 after PRP (0.39 ± 0.12 logMAR, *p* = 0.032) compared to baseline, as it is shown in Table 2. 

At baseline, the mean CST was 262.4 ± 35.2 μm. There was a statistically significant increase in CST at month 1 after PRP compared to baseline (299.1 ± 42.7 μm, *p* < 0.001), while CST did not differ at month 6 (274.3 ± 37.7 μm, *p* = 0.228) and month 12 (270.1 ± 34.1 μm, *p* = 0.409) after PRP compared to baseline, as it is shown in Table 2. 

In addition, the ellipsoid zone was intact in 20 out of 28 patients (71.4%) at baseline. There was no statistically significant change in ellipsoid zone condition at month 1 (*p* = 0.763), month 6 (*p* = 0.537), and month 12 (*p* = 0.537) of the follow-up period, as it is illustrated in Table 2. Accordingly, at baseline, the external limiting membrane was intact in 22 out of 28 patients (78.6%), and remained the same throughout the whole follow-up period. 

Table 3 shows the OCTA parameters in SCP and DCP at baseline and at the 1-month, 6-month and 12-month follow up after PRP. At baseline, the mean FAZ area was found to be 0.51 ± 0.18 mm^2^, with a 6.43 ± 2.41 mm perimeter and 0.21 ± 0.09 circularity ratio. There was a statistically significant reduction in the FAZ area at month 6 (0.402 ± 0.13 mm^2^, *p* = 0.014) and month 12 (0.406 ± 0.10 mm^2^, *p* = 0.011) after PRP compared to baseline. Accordingly, at months 6 and 12, the FAZ perimeter was significantly lower compared to baseline (*p* = 0.015 and *p* = 0.017 for month 6 and 12, respectively). In addition, FAZ became more circular at month 6 (0.27 ± 0.08, *p* = 0.009) and month 12 (0.27 ± 0.10, *p* = 0.015) after PRP compared to baseline. 

There was a statistically significant increase in the mean central VD at the SCP at month 6 (*p* = 0.002) and month 12 (*p* = 0.003) after PRP compared to baseline. In addition to the foveal area, the mean VD was significantly increased in the parafoveal area at all quadrants at the SCP at months 6 and 12 after PRP compared to baseline, as it is shown in Table 3. Regarding the DCP, no statistically significant difference was observed in foveal and parafoveal VD at any quadrant and at any time-point of the follow-up period compared to baseline, as it is depicted in Table 3. 

## 4. Discussion

In the present study, we investigated the alterations in OCTA parameters after PRP in patients with PDR. The principal message that came from our study is that there was a statistically significant decrease in FAZ area 6 months after PRP for PDR compared to baseline, which remained until the end of our observation, at month 12 after PRP. Additionally, a significant increase in the foveal and parafoveal VD at SCP was found at month 6 and 12 of the follow-up period. However, although VD and FAZ area were improved, there was no statistically significant difference in BCVA over time compared to baseline.

Previous studies have investigated ocular blood flow, and tried to obtain quantitative data on retinal and choroidal circulation in eyes with DR after PRP, using various methods, such as colour Doppler imaging, laser interferometry, laser Doppler flowmetry and laser speckle flowgraphy [8,16,17,18,19,20,21]. Augsten et al. found that PRP improved the choroidal circulation in the macular area in patients with NPDR, using a reflection spectra method [19]. Takahashi et al. also reported a significant increase in subfoveal choroidal flow one month after PRP in patients without macular oedema, using laser doppler flowmetry [20]. They hypothesised that the redistribution of choroidal flow from obliterated peripheral capillaries to the macula and the inflammatory response to PRP were the main underlying mechanisms of macular choroidal flow increase [20]. These findings were in accordance with those of preclinical studies, since Flower et al. implied that PRP markedly increased the choriocapillaris blood flow in the macular area compared to the periphery in monkeys after the coagulation of the peripheral retina, using indocyanine green angiography [21]. 

On the other hand, several researchers tried to examine changes in VD after PRP in patients with PDR using OCTA, which can provide a non-invasive and more reliable assessment of retinal blood flow. Lorusso et al. did not find any significant change in the retinal haemodynamics indices at 6-month follow up after laser photocoagulation using a frequency-doubled Nd:YAG laser instead of an argon laser for PDR, and including patients with both type 1 and type 2 DM, which could have affected their results [12]. Similarly, Zhao et al. reported no change in OCTA parameters after PRP in eyes with PDR or severe NPDR, in a study with limited follow up, ranging from 1–3 months [22]. Moreover, Fawzi et al. did not find a significant change in macular VD following PRP in eyes with PDR, although the adjusted flow index (a self-created surrogate metric of blood flow) suggested an overall redistribution of blood flow to the posterior pole, with the improvement of capillary flow after PRP [14]. On the contrary, Kim et al. demonstrated a decrease in VD one month after PRP in eyes with PDR or severe NPDR compared to baseline findings, while they observed a significant increase in VD at both SCP and DCP 12 months after PRP [23]. Very similar results were found by Mirshahi et al., who showed a significant increase in VD in both SCP and DCP 3 months after PRP in 11 eyes with PDR or severe NPDR [13].

The exact mechanism of the early decrease in VD following PRP is still unknown. One possible explanation for this reduction is that the capillary flow following PRP might be associated with early PRP-induced inflammation and nitric oxide (NO) overproduction. After a sufficient time, the inflammation probably subsides, and subsequently, autoregulatory functions of retinal vasculature may occur, leading to an improvement in VD at the long-term follow up [23]. Furthermore, the main theory about the mechanism of action of PRP pertains to the fact that the retinal pigment epithelium (RPE) absorbs the laser energy, and, consequently, thermal energy is generated in the outer retina, destroying the peripheral RPE and adjacent photoreceptors to the reduced retinal oxygen consumption [13,14]. Due to this, it has been assumed that the redistribution of blood flow from the periphery to the macular region after PRP results in the re-organisation of capillary networks, resulting in the better oxygenation of retina and a consequent decrease in VEGF levels and increase in VD [23]. 

Regarding the FAZ alterations after PRP, the results of the existing studies are controversial. The enlargement and irregularity of the FAZ are important indications of diabetic macular ischemia [24,25,26]. Salz et al. have found that patients with PDR have a larger FAZ compared to normal eyes, and enlargement of the FAZ indicates an increase in non-perfusion, since the progression of DR may be associated with a more irregular FAZ due to capillary occlusion or altered blood flow [27]. Lorusso et al. found no change in FAZ six months after PRP treatment in PDR eyes [12], in line with the results of Misharhi et al., who also found no significant changes in the FAZ area three months following PRP [13]. On the other hand, Sabaner et al. reported a significant decrease in FAZ area after PRP in NPDR eyes [28], while Abdelhalim et al. presented a significant decrease in FAZ area 6 months after PRP in PDR [29]. Faghihi et al. also showed that the FAZ area constricted and became significantly more circular at the 6-month follow up after PRP [30], as we found in our study at the long-term follow up of 12 months. The latter could be explained by the fact that improved and more effective flow in the remaining capillaries of the macular area could result in the more effective perfusion of the posterior pole [30]. Of note is that the discrepancy between the results of various studies could be attributed to the different OCTA machines and software used, as well as the difference in population and follow up of each study. 

Another important finding of our study was that the BCVA remained the same throughout the 12-month follow-up period, although there was an improvement in foveal and parafoveal VD at SCP. One could hypothesise that the functional improvement may need more time than the structural one, suggesting that improvement in visual acuity may occur a long time after the improvement in VD. In addition, there was no statistically significant difference in the VD at the DCP over time, and this could also have an impact on visual acuity. However, it should be noted that visual acuity is mainly affected by the ellipsoid zone condition [31,32].

A potential limitation of our study pertains to the lack of a control group, which prevents us from drawing a firm conclusion regarding the association between changes in OCTA parameters and PRP treatment. However, it is unethical not to treat patients with PDR, and thus the use of a control group is not feasible. In addition, the lack of considerations of other foveal area hemodynamic parameters, such as choroidal VD and fractal dimensions, could be added as a limitation of the study. Moreover, multivariate analysis taking into account HbA1c levels, hypertension, dyslipidaemia, and demographic data was not performed, since we lack of data at month 12, as they were not included in the protocol of the study. It is worth noting, however, that this is a prospective study with a relatively large sample size of 28 patients and a long-term follow up of 12 months, both considerable in comparison to previous studies, while we included only patients with high-risk PDR without diabetic macular oedema, so as to have a homogenous population. In addition, we conducted the first study in the literature on the ellipsoid zone condition in association with OCTA parameters in patients with PDR after PRP.

## 5. Conclusions

In conclusion, our study revealed that there was a significant improvement in foveal and parafoveal VD at the SCP at the 6- and 12-month follow up after PRP in patients with PDR without macular oedema. The increase in VD suggests that a redistribution of ocular blood flow may occur from the peripheral retina to the centre after PRP, leading to the better oxygenation of the macular area. This assumption may be supported by the fact that the FAZ area was restored 12 months after PRP, presenting better circularity and decreased FAZ area compared to the baseline condition. However, although VD at SCP and FAZ area were improved, the visual acuity was found to be the same throughout the follow up. Further studies with a larger sample size and better OCTA software are needed to corroborate our results. 

## Figures and Tables

**Table 1:** Demographic and clinical characteristics of the study sample.

Age (years, mean ± SD) (min–max)	57.3 ± 8.9 (48–68)
Gender (n, %)	
Male	15 (53.6%)
Female	13 (46.4%)
Duration of diabetes mellitus (years, mean ± SD) (min–max)	12.4 ± 5.4 (7–19)
HbA1c (%, mean ± SD) (min–max)	7.8 ± 1.3 (6–10.5)
Hypertension (n, %)	18 (64.3%)
Dyslipidemia (n, %)	12 (42.9%)
Smoking (n, %)	11 (39.2%)
Best-corrected visual acuity (logMAR, mean ± SD) (min–max)	0.41 ± 0.11 (0.3–0.6)
Intraocular pressure (mmHg, mean ± SD) (min–max)	15.7 ± 4.2 (11–18)
Lens status (n, %)	
Phakic	22 (78.6%)
Pseudophakic	6 (21.4%)
Refractive error (spherical equivalent in D, mean ± SD)	−1.25 ± 0.75
Central subfield thickness (μm, mean ± SD) (min–max)	262.4 ± 35.2 (223–309)
Ellipsoid zone condition (n, %)	
Intact	20 (71.4%)
Disrupted	8 (28.6%)
External limiting membrane condition (n, %)	
Intact	22 (78.6%)
Disrupted	6 (21.4%)

**Table 2:** Changes in visual acuity, central subfield thickness, ellipsoid zone, and external limiting membrane over time.

	Baseline	Month 1	Month 6	Month 12
Best-corrected visual acuity (logMAR, mean ± SD) (min–max)	0.41 ± 0.11 (0.3–0.6)	0.41 ± 0.13 (0.3–0.6) (*p* = 0.030)	0.40 ± 0.12 (0.3–0.6) (*p* = 0.103)	0.39 ± 0.12 (0.3–0.6) (*p* = 0.032)
Central subfield thickness (μm, mean ± SD) (min–max)	262.4 ± 35.2 (223–309)	299.1 ± 42.7 (242–350) (*p* < 0.001)	274.3 ± 37.7 (231–315) (*p* = 0.228)	270.1 ± 34.1 (232–311) (*p* = 0.409)
Ellipsoid zone integrity intact (n, %)	20/28 (71.4%)	21/28 (75%) (*p* = 0.763)	22/28 (78.6%) (*p* = 0.537)	22/28 (78.6%) (*p* = 0.537)
External limiting membrane integrity intact (n, %)	22/28 (78.6%)	22/28 (78.6%)	22/28 (78.6%)	22/28 (78.6%)

**Table 3:** Optical coherence tomography angiography findings at baseline and at months 1, 6, and 12 after panretinal photocoagulation. Bold denotes statistical significance.

	Baseline	Month 1	Month 6	Month 12
FAZ area (mm^2^, mean ± SD)	0.51 ± 0.18	0.51 ± 0.19 (*p* = 0.952)	0.40 ± 0.13 **(*p* = 0.014)**	0.41 ± 0.10 **(*p* = 0.011)**
FAZ perimeter (mm, mean ± SD)	6.43 ± 2.41	6.45 ± 2.03 (*p* = 0.973)	5.02 ± 1.73 **(*p* = 0.015)**	5.01 ± 1.88 **(*p* = 0.017)**
FAZ circularity (mean ± SD)	0.21 ± 0.09	0.21 ± 0.10 (*p* > 0.999)	0.27 ± 0.08 **(*p* = 0.009)**	0.27 ± 0.10 **(*p* = 0.015)**
**Superficial capillary plexus (mean ± SD)**				
Vessel density, central	9.07 ± 4.92	10.91 ± 4.01 (*p* = 0.102)	12.96 ± 4.06 **(*p* = 0.002)**	13.15 ± 4.89 **(*p* = 0.003)**
Vessel density, superior	26.05 ± 10.21	30.97 ± 7.17 (*p* = 0.028)	32.21 ± 7.56 **(*p* = 0.013)**	32.45 ± 7.74 **(*p* = 0.010)**
Vessel density, inferior	24.71 ± 11.63	26.95 ± 8.14 (*p* = 0.289)	31.99 ± 7.85 **(*p* = 0.008)**	32.12 ± 7.76 **(*p* = 0.007)**
Vessel density, temporal	19.52 ± 9.35	24.96 ± 9.55 (*p* = 0.036)	25.67 ± 9.40 **(p = 0.017)**	26.13 ± 9.42 **(*p* = 0.010)**
Vessel density, nasal	22.15 ± 10.61	27.13 ± 9.23 (*p* = 0.067)	28.77 ± 9.06 **(*p* = 0.015)**	28.93 ± 9.57 **(*p* = 0.015)**
**Deep capillary plexus (mean ± SD)**				
Vessel density, central	5.01 ± 2.73	5.12 ± 2.96 (*p* = 0.886)	6.31 ± 2.57 (*p* = 0.059)	6.69 ± 3.17 (*p* = 0.038)
Vessel density, superior	12.99 ± 6.81	14.73 ± 6.62 (*p* = 0.337)	16.57 ± 7.59 (*p* = 0.069)	16.96 ± 6.53 (*p* = 0.030)
Vessel density, inferior	15.61 ± 5.53	16.06 ± 6.12 (*p* = 0.774)	17.89 ± 6.41 (*p* = 0.160)	18.15 ± 5.94 (*p* = 0.104)
Vessel density, temporal	16.11 ± 4.90	17.03 ± 4.51 (*p* = 0.468)	17.98 ± 4.99 (*p* = 0.163)	19.29 ± 5.23 (*p* = 0.023)
Vessel density, nasal	15.17 ± 3.78	15.88 ± 3.91 (*p* = 0.493)	16.91 ± 4.07 (*p* = 0.103)	17.52 ± 4.90 (*p* = 0.050)

## Data Availability

Data will be made available upon request.

## References

[1] International Diabetes Federation (2021). IDF Diabetes Atlas.

[2] Antonetti D.A., Klein R., Gardner T.W. (2012). Diabetic retinopathy. N. Engl. J. Med..

[3] The Diabetic Retinopathy Study Research Group (1981). Photocoagulation treatment of proliferative diabetic retinopathy. Clinical application of Diabetic Retinopathy Study (DRS) findings, DRS Report Number 8. Ophthalmology.

[4] Funatsu H., Hori S., Yamashita H., Kitano S. (1996). Effective mechanisms of laser photocoagulation for neovascularization in diabetic retinopathy. Nippon. Ganka Gakkai Zasshi.

[5] Luttrull J.K. (2023). Lasers in Medicine: The Changing Role of Therapeutic Laser-Induced Retinal Damage—From de rigeuer to Nevermore. Photonics.

[6] Moriarty A.P., Spalton D.J., Shilling J.S., Ffytche T.J., Bulsara M. (1996). Breakdown of the blood-aqueous barrier after argon laser panretinal photocoagulation for proliferative diabetic retinopathy. Ophthalmology.

[7] Mendivil A. (1997). Ocular blood flow velocities in patients with proliferative diabetic retinopathy after panretinal photocoagulation. Surv. Ophthalmol..

[8] Yamada Y., Suzuma K., Onizuka N., Uematsu M., Mohamed Y.H., Kitaoka T. (2017). Evaluation of retinal blood flow before and after panretinal photocoagulation using pattern scan laser for diabetic retinopathy. Curr. Eye Res..

[9] Iwase T., Kobayashi M., Yamamoto K., Ra E., Terasaki H. (2017). Effects of photocoagulation on ocular blood flow in patients with severe non-proliferative diabetic retinopathy. PLoS ONE.

[10] Cicinelli M.V., Cavalleri M., Brambati M., Lattanzio R., Bandello F. (2019). New imaging systems in diabetic retinopathy. Acta Diabetol..

[11] Russell J.F., Al-Khersan H., Shi Y., Scott N.L., Hinkle J.W., Fan K.C., Lyu C., Feuer W.J., Gregori G., Rosenfeld P.J. (2020). Retinal Nonperfusion in Proliferative Diabetic Retinopathy Before and After Panretinal Photocoagulation Assessed by Widefield OCT Angiography. Am. J. Ophthalmol..

[12] Lorusso M., Milano V., Nikolopoulou E., Ferrari L.M., Cicinelli M.V., Querques G., Ferrari T.M. (2019). Panretinal Photocoagulation Does Not Change Macular Perfusion in Eyes With Proliferative Diabetic Retinopathy. Ophthalmic Surg. Lasers Imaging Retin..

[13] Mirshahi A., Ghassemi F., Fadakar K., Mirshahi R., Bazvand F., Riazi-Esfahani H. (2019). Effects of panretinal photocoagulation on retinal vasculature and foveal avascular zone in diabetic retinopathy using optical coherence tomography angiography: A pilot study. J. Curr. Ophthalmol..

[14] Fawzi A.A., Fayed A.E., Linsenmeier R.A., Gao J., Yu F. (2019). Improved Macular Capillary Flow on Optical Coherence Tomography Angiography after Panretinal Photocoagulation for Proliferative Diabetic Retinopathy. Am. J. Ophthalmol..

[15] Early Treatment Diabetic Retinopathy Study Research Group (1991). Grading diabetic retinopathy from stereoscopic color fundus photographs—An extension of the modified Airlie House classification. ETDRS report number 10. Ophthalmology.

[16] Lee J.C., Wong B.J., Tan O., Srinivas S., Sadda S.R., Huang D., Fawzi A.A. (2013). Pilot study of Doppler optical coherence tomography of retinal blood flow following laser photocoagulation in poorly controlled diabetic patients. Investig. Ophthalmol. Vis. Sci..

[17] Grunwald J.E., Brucker A.J., Grunwald S.E., Riva C.E. (1993). Retinal hemodynamics in proliferative diabetic retinopathy. A laser Doppler velocimetry study. Investig. Ophthalmol. Vis. Sci..

[18] Feke G.T., Green G.J., Goger D.G., McMeel J.W. (1982). Laser Doppler measurements of the effect of panretinal photocoagulation on retinal blood flow. Ophthalmology.

[19] Augsten R., Königsdörffer E., Schweitzer D., Strobel J. (1998). Nonproliferative diabetic retinopathy-reflection spectra of the macula before and after laser photocoagulation. Ophthalmologica.

[20] Takahashi A., Nagaoka T., Sato E., Yoshida A. (2008). Effect of panretinal photocoagulation on choroidal circulation in the foveal region in patients with severe diabetic retinopathy. Br. J. Ophthalmol..

[21] Flower R.W., Fryczkowski A.W., McLeod D.S. (1995). Variability in choriocapillaris blood flow distribution. Investig. Ophthalmol. Vis. Sci..

[22] Zhao T., Chen Y., Liu D., Stewart J.M. (2020). Optical Coherence Tomography Angiography Assessment of Macular Choriocapillaris and Choroid Following Panretinal Photocoagulation in a Diverse Population With Advanced Diabetic Retinopathy. Asia Pac. J. Ophthalmol..

[23] Kim K., Kim E.S., Yu S.Y. (2021). Longitudinal changes in retinal microvasculature after panretinal photocoagulation in diabetic retinopathy using swept-source OCT angiography. Sci. Rep..

[24] Takase N., Nozaki M., Kato A., Ozeki H., Yoshida M., Ogura Y. (2015). Enlargement of foveal avascular zone in diabetic eyes evaluated by en face optical coherence tomography angiography. Retina.

[25] Early Treatment Diabetic Retinopathy Study Research Group (1991). Classification of diabetic retinopathy from fluorescein angiograms. ETDRS report number 11. Ophthalmology.

[26] Krawitz B.D., Mo S., Geyman L.S., Agemy S.A., Scripsema N.K., Garcia P.M., Chui T.Y., Rosen R.B. (2017). Acircularity index and axis ratio of the foveal avascular zone in diabetic eyes and healthy controls measured by optical coherence tomography angiography. Vis. Res..

[27] Salz D.A., de Carlo T.E., Adhi M., Moult E., Choi W., Baumal C.R., Witkin A.J., Duker J.S., Fujimoto J.G., Waheed N.K. (2016). Select Features of Diabetic Retinopathy on Swept-Source Optical Coherence Tomographic Angiography Compared with Fluorescein Angiography and Normal Eyes. JAMA Ophthalmol..

[28] Sabaner M.C., Dogan M., Akdogan M., Şimşek M. (2021). Panretinal laser photocoagulation decreases large foveal avascular zone area in non-proliferative diabetic retinopathy: A prospective OCTA study. Photodiagnosis Photodyn. Ther..

[29] Abdelhalim A.S., Abdelkader M.F.S.O., Mahmoud M.S.E.-D., Mohamed Mohamed A.A. (2022). Macular vessel density before and after panretinal photocoagulation in patients with proliferative diabetic retinopathy. Int. J. Retin. Vitr..

[30] Faghihi H., Riazi-Esfahani H., Khodabande A., Khalili Pour E., Mirshahi A., Ghassemi F., Mirshahi R., Khojasteh H., Bazvand F., Hashemi A. (2021). Effect of panretinal photocoagulation on macular vasculature using optical coherence tomography angiography. Eur. J. Ophthalmol..

[31] Theodossiadis P.G., Theodossiadis G.P., Charonis A., Emfietzoglou I., Grigoropoulos V.G., Liarakos V.S. (2011). The photoreceptor layer as a prognostic factor for visual acuity in the secondary epiretinal membrane after retinal detachment surgery: Imaging analysis by spectral-domain optical coherence tomography. Am. J. Ophthalmol..

[32] Chatziralli I., Theodossiadis G., Dimitriou E., Kazantzis D., Theodossiadis P. (2020). Association between the patterns of diabetic macular edema and photoreceptors’ response after intravitreal ranibizumab treatment: A spectral-domain optical coherence tomography study. Int. Ophthalmol..

